# Supplementary material for: A Humanized Yeast Phenomic Model of Deoxycytidine Kinase to Predict Genetic Buffering of Nucleoside Analog Cytotoxicity
Source: Genes (Basel). 2019 Sep 30;10(10):770. doi: 10.3390/genes10100770 (PMC6826991; doi:10.3390/genes10100770)
Supplement: Supplementary file 1 [file genes-10-00770-s001.zip › Additional_Files/Additional_File7_GOTermHeatmaps.zip!/Additional_File7_GOTermHeatmaps/double-strand_break_repair.pdf]

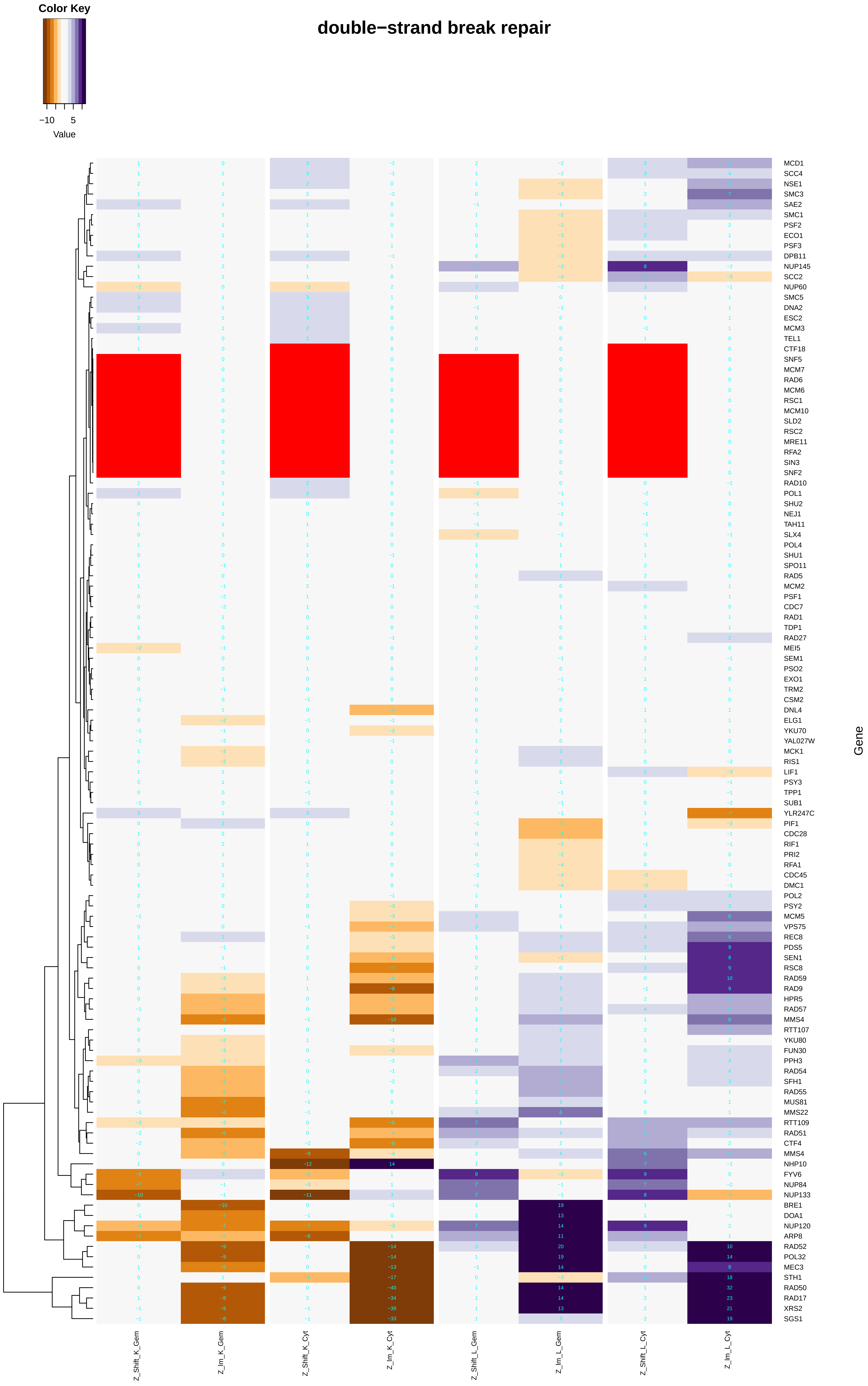
double−strand break repair
Color Key
−10
5
Value
Gene
MCD1
SCC4
NSE1
SMC3
SAE2
SMC1
PSF2
ECO1
PSF3
DPB11
NUP145
SCC2
NUP60
SMC5
DNA2
ESC2
MCM3
TEL1
CTF18
SNF5
MCM7
RAD6
MCM6
RSC1
MCM10
SLD2
RSC2
MRE11
RFA2
SIN3
SNF2
RAD10
POL1
SHU2
NEJ1
TAH11
SLX4
POL4
SHU1
SPO11
RAD5
MCM2
PSF1
CDC7
RAD1
TDP1
RAD27
MEI5
SEM1
PSO2
EXO1
TRM2
CSM2
DNL4
ELG1
YKU70
YAL027W
MCK1
RIS1
LIF1
PSY3
TPP1
SUB1
YLR247C
PIF1
CDC28
RIF1
PRI2
RFA1
CDC45
DMC1
POL2
PSY2
MCM5
VPS75
REC8
PDS5
SEN1
RSC8
RAD59
RAD9
HPR5
RAD57
MMS4
RTT107
YKU80
FUN30
PPH3
RAD54
SFH1
RAD55
MUS81
MMS22
RTT109
RAD51
CTF4
MMS4
NHP10
FYV6
NUP84
NUP133
BRE1
DOA1
NUP120
ARP8
RAD52
POL32
MEC3
STH1
RAD50
RAD17
XRS2
SGS1
Z_Shift_K_Gem
Z_Im_K_Gem
Z_Shift_K_Cyt
Z_Im_K_Cyt
Z_Shift_L_Gem
Z_Im_L_Gem
Z_Shift_L_Cyt
Z_Im_L_Cyt

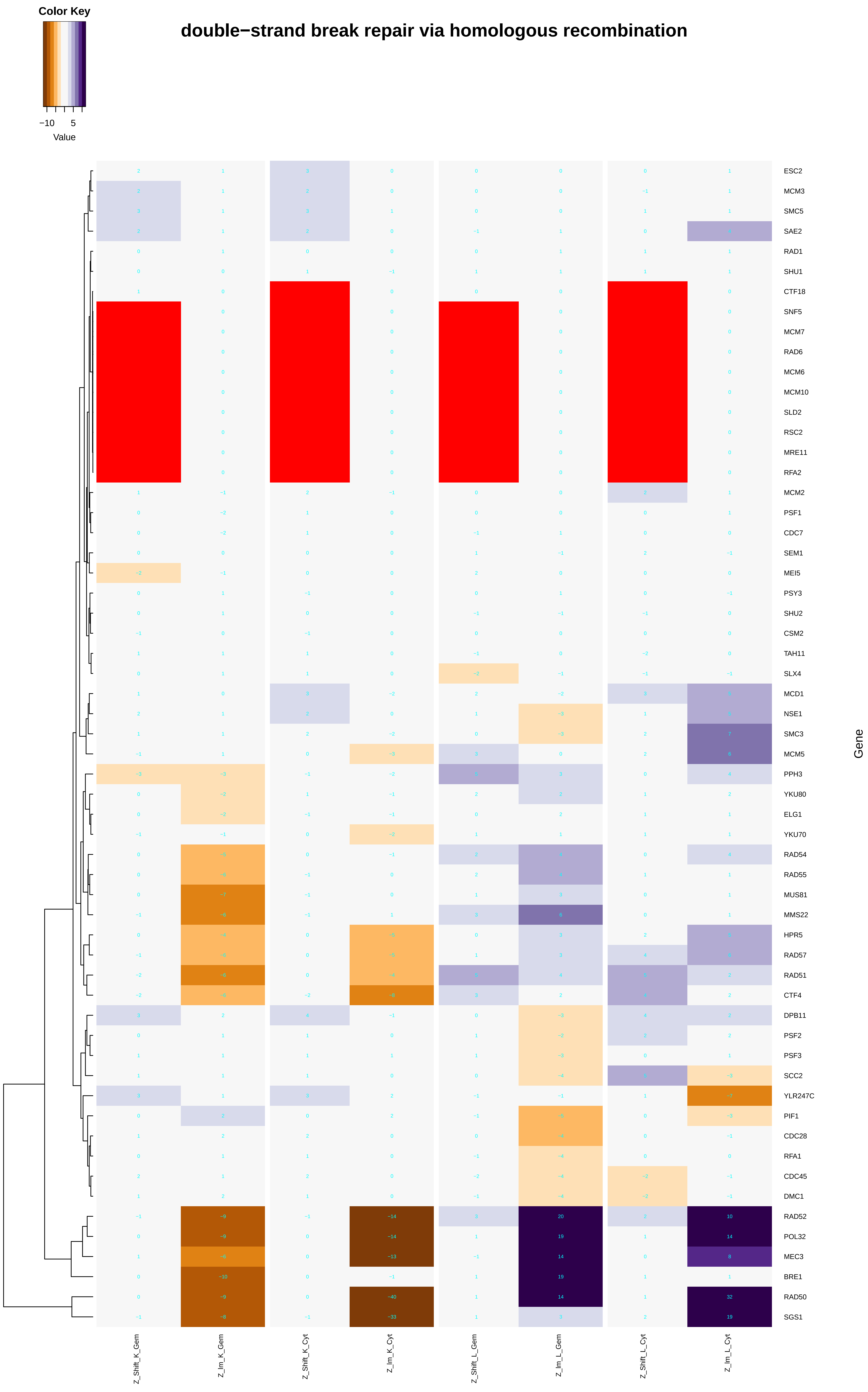
Color Key
−10 5
Value
double−strand break repair via homologous recombination
ESC2
MCM3
SMC5
SAE2
RAD1
SHU1
CTF18
SNF5
MCM7
RAD6
MCM6
MCM10
SLD2
RSC2
MRE11
RFA2
MCM2
PSF1
CDC7
SEM1
MEI5
PSY3
SHU2
CSM2
TAH11
SLX4
MCD1
NSE1
SMC3
MCM5
PPH3
YKU80
ELG1
YKU70
RAD54
RAD55
MUS81
MMS22
HPR5
RAD57
RAD51
CTF4
DPB11
PSF2
PSF3
SCC2
YLR247C
PIF1
CDC28
RFA1
CDC45
DMC1
RAD52
POL32
MEC3
BRE1
RAD50
SGS1
Gene
Z_Shift_K_Gem
Z_Im_K_Gem
Z_Shift_K_Cyt
Z_Im_K_Cyt
Z_Shift_L_Gem
Z_Im_L_Gem
Z_Shift_L_Cyt
Z_Im_L_Cyt

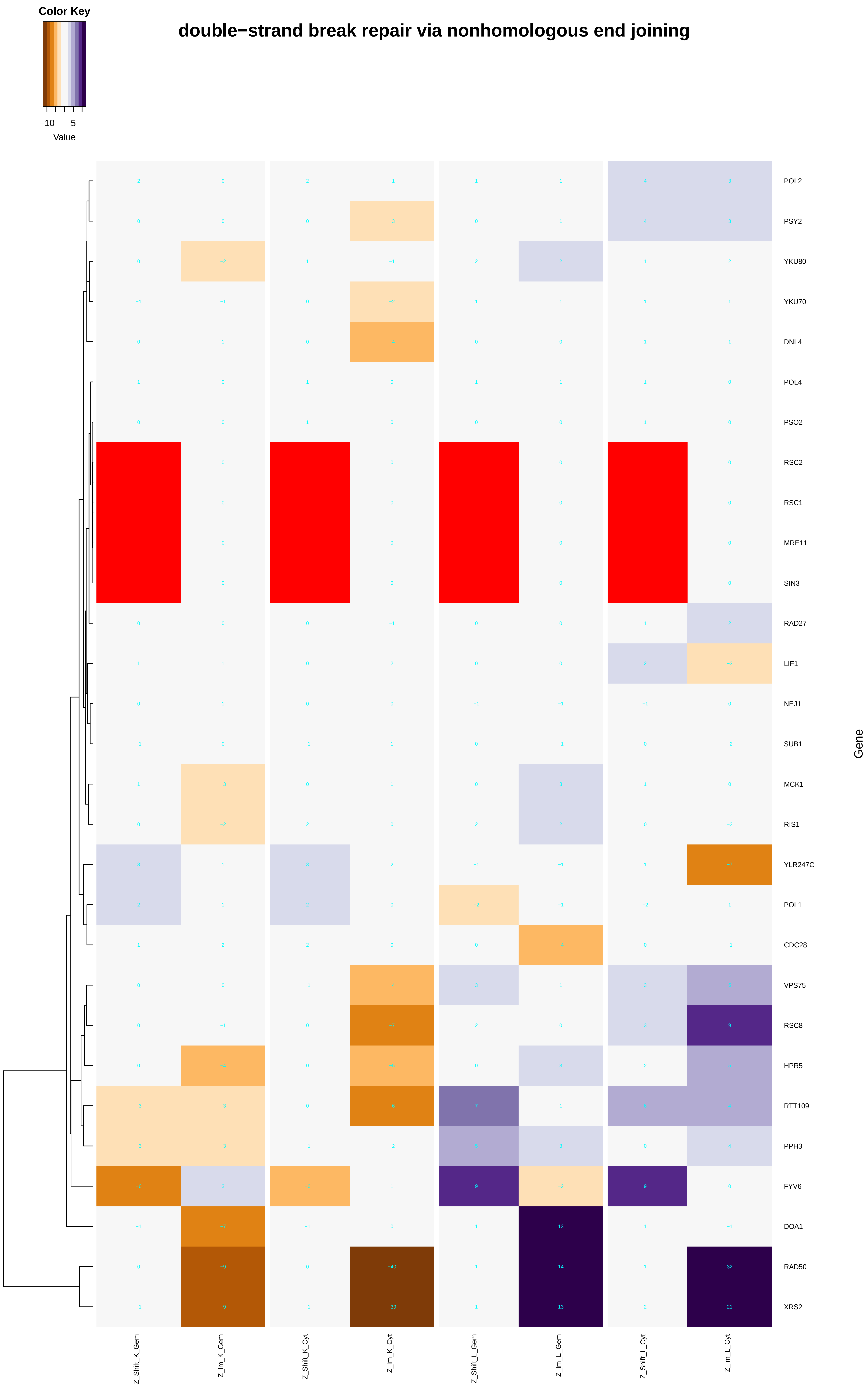

Color Key
−10
5
Value
double−strand break repair via nonhomologous end joining
POL2
PSY2
YKU80
YKU70
DNL4
POL4
PSO2
RSC2
RSC1
MRE11
SIN3
RAD27
LIF1
NEJ1
SUB1
MCK1
RIS1
YLR247C
POL1
CDC28
VPS75
RSC8
HPR5
RTT109
PPH3
FYV6
DOA1
RAD50
XRS2
Gene
Z_Shift_K_Gem
Z_Im_K_Gem
Z_Shift_K_Cyt
Z_Im_K_Cyt
Z_Shift_L_Gem
Z_Im_L_Gem
Z_Shift_L_Cyt
Z_Im_L_Cyt

double−strand break repair via single−strand annealing

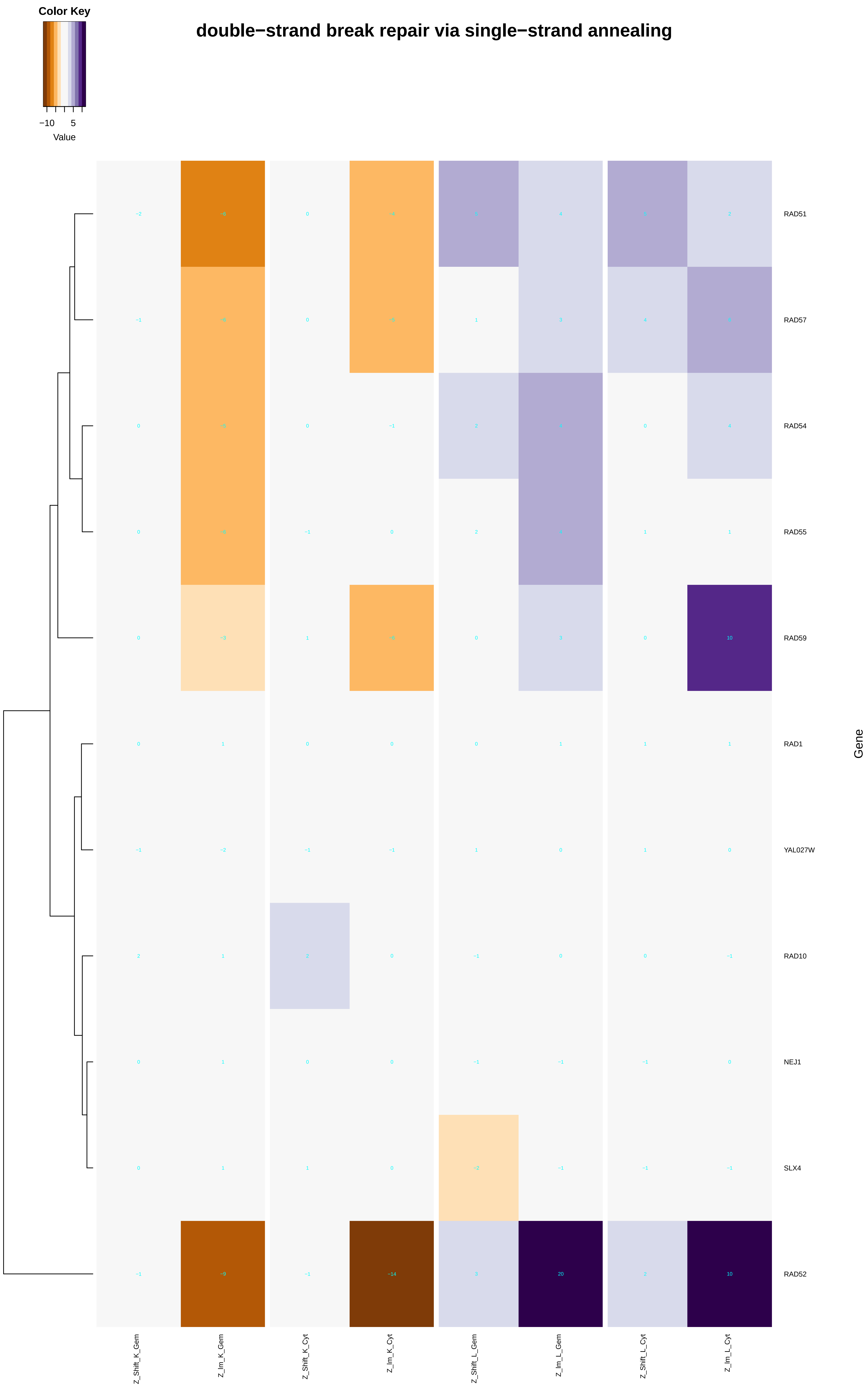

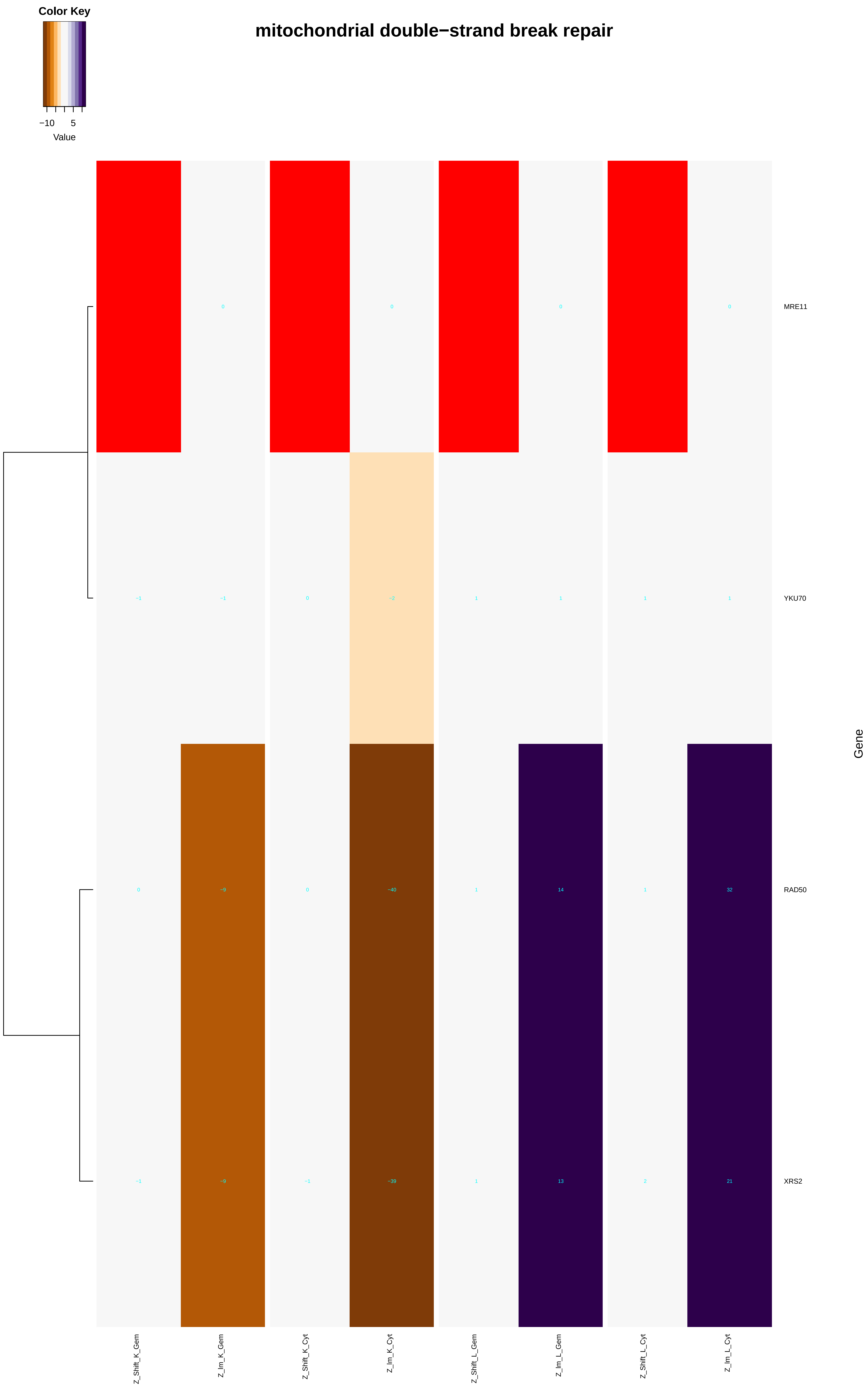

Color Key
−10
5
Value
mitochondrial double−strand break repair
MRE11
YKU70
RAD50
XRS2
Gene
Z_Shift_K_Gem
Z_Im_K_Gem
Z_Shift_K_Cyt
Z_Im_K_Cyt
Z_Shift_L_Gem
Z_Im_L_Gem
Z_Shift_L_Cyt
Z_Im_L_Cyt

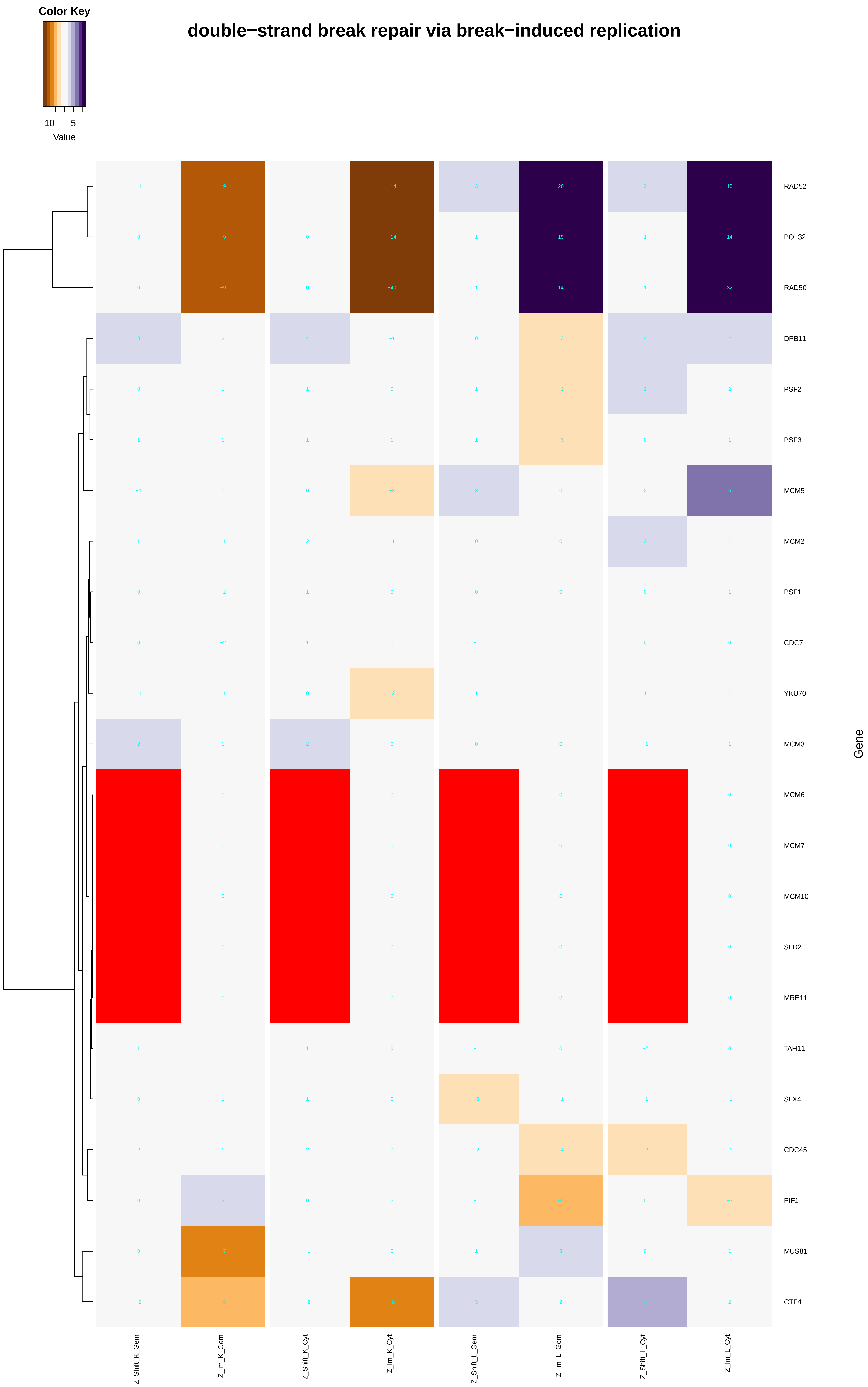

double−strand break repair via break−induced replication
Color Key
−10
5
Value
RAD52
POL32
RAD50
DPB11
PSF2
PSF3
MCM5
MCM2
PSF1
CDC7
YKU70
MCM3
MCM6
MCM7
MCM10
SLD2
MRE11
TAH11
SLX4
CDC45
PIF1
MUS81
CTF4
Gene
Z_Shift_K_Gem
Z_Im_K_Gem
Z_Shift_K_Cyt
Z_Im_K_Cyt
Z_Shift_L_Gem
Z_Im_L_Gem
Z_Shift_L_Cyt
Z_Im_L_Cyt

double−strand break repair via synthesis−dependent strand annealing

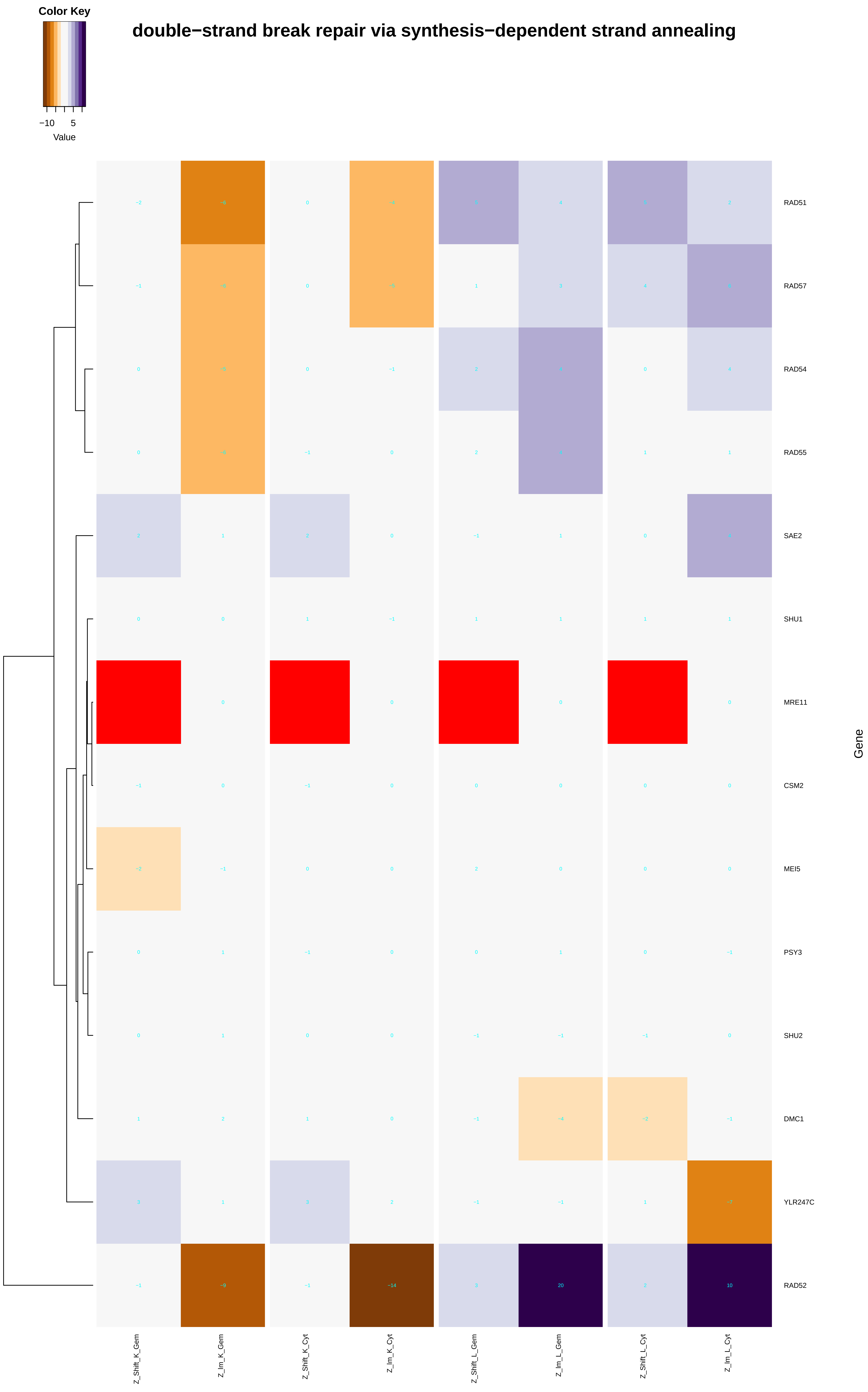

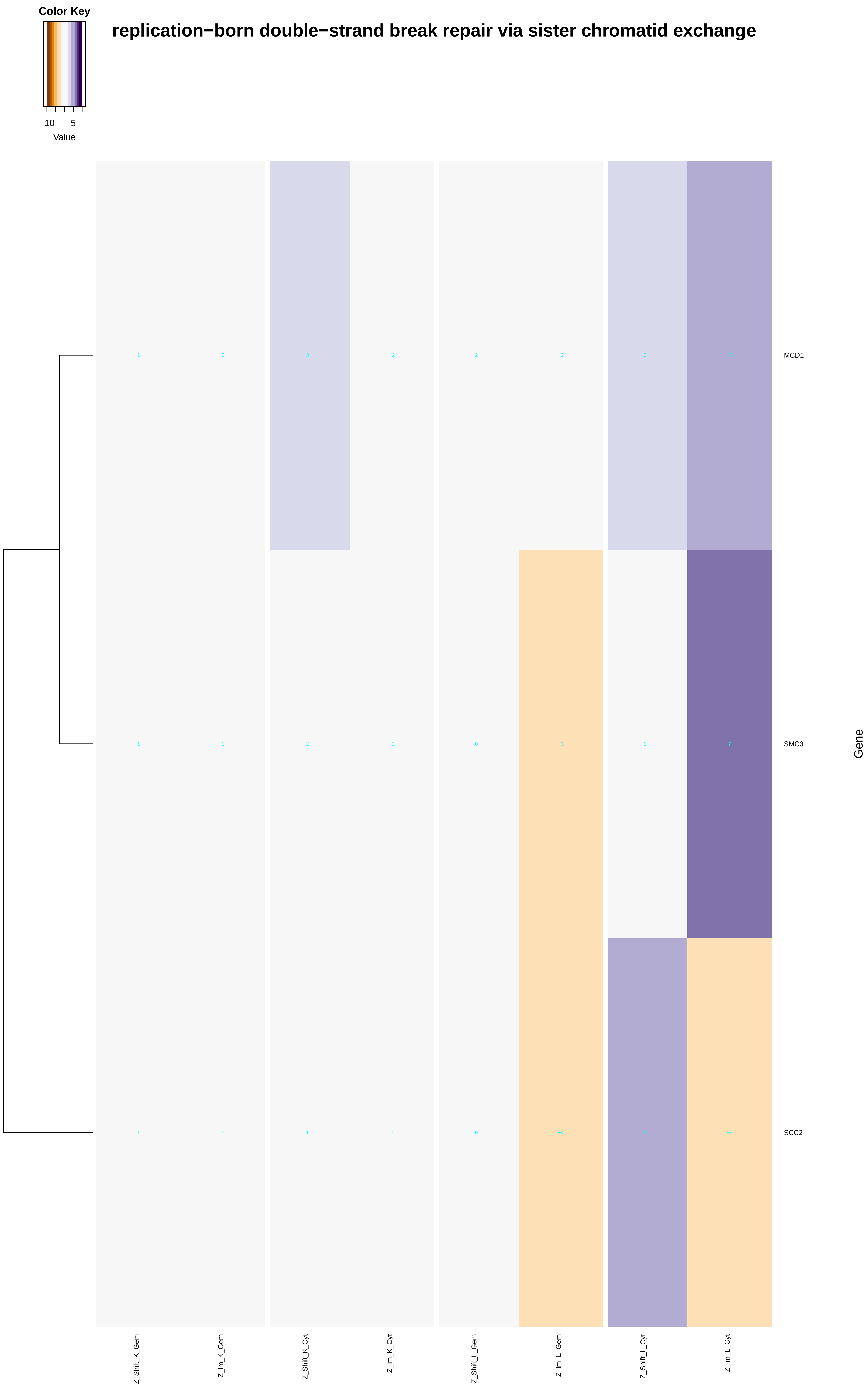
replication−born double−strand break repair via sister chromatid exchange
Color Key
−10
5
Value
MCD1
SMC3
SCC2
Gene
Z_Shift_K_Gem
Z_lm_K_Gem
Z_Shift_K_Cyt
Z_lm_K_Cyt
Z_Shift_L_Gem
Z_lm_L_Gem
Z_Shift_L_Cyt
Z_lm_L_Cyt

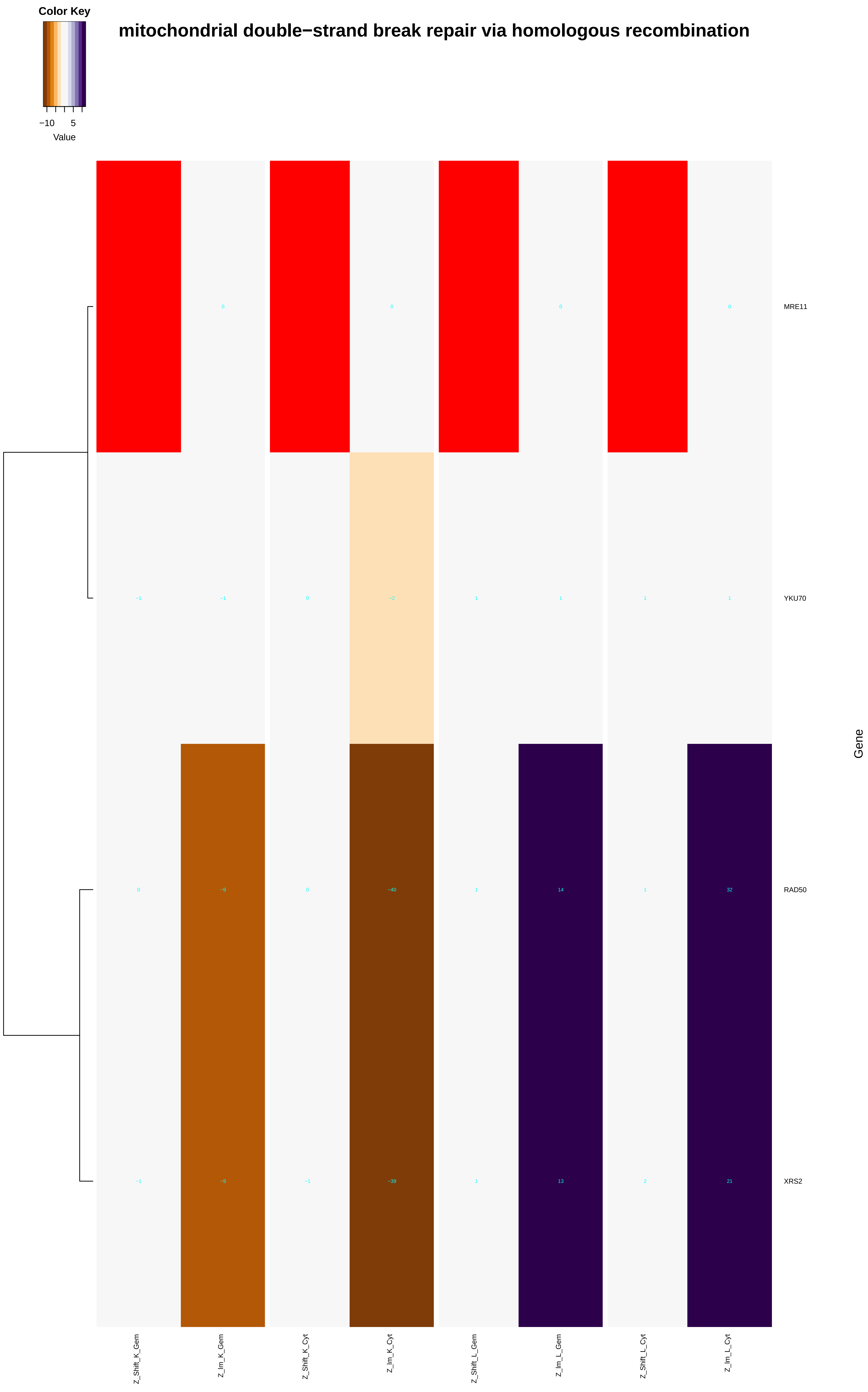

Color Key
−10 5
Value
mitochondrial double−strand break repair via homologous recombination
MRE11
YKU70
RAD50
XRS2
Gene
Z_Shift_K_Gem
Z_Im_K_Gem
Z_Shift_K_Cyt
Z_Im_K_Cyt
Z_Shift_L_Gem
Z_Im_L_Gem
Z_Shift_L_Cyt
Z_Im_L_Cyt
